# The effectiveness of psychological interventions in elite sport: methodological issues and opportunities to gather evidence

**DOI:** 10.3389/fpsyg.2025.1516760

**Published:** 2025-01-29

**Authors:** Marjorie Bernier, Pierre Bagot, Nadia Sondt, Guillaume Levillain, Philippe Vacher, Julie Doron, Guillaume Martinent, Jean F. Fournier, Gilles Kermarrec

**Affiliations:** ^1^Univ Brest, Centre de Recherche sur l'Education les Apprentissages et la Didactique, CREAD, EA 3875, Brest, France; ^2^Complexité, Innovation, Activités Motrices et Sportives (CIAMS), Paris-Saclay University, Orsay, France; ^3^Complexité, Innovation, Activités Motrices et Sportives (CIAMS), Orleans University, Orléans, France; ^4^Sport, Activité Physique, Rééducation et Motricité (SAPRéM), Orleans University, Orléans, France; ^5^Nantes Université, Movement - Interactions - Performance, MIP, UR 4334, Nantes, France; ^6^Laboratoire sur les Vulnérabilités et l'Innovation dans le Sport (EA 7428), Université Claude Bernard Lyon 1 – Université de Lyon, Lyon, France; ^7^Université Paris Nanterre, Laboratoire Interdisciplinaire en Neurosciences, Physiologie et Psychologie (LINP2), Nanterre, France

**Keywords:** elite sport, psychological interventions, evidence, methods, performance

## 1 Introduction

The evaluation of the effects of sport psychology interventions on performance and related outcomes is a central issue in evidence-based practice. Although there is an extensive literature in the domain of sport psychology, recent meta-analyses have only identified a few interventional studies that test the effects of different psychological interventions on performance (Brown and Fletcher, 2017; Lochbaum et al., 2022). While Brown and Fletcher (2017) identified 35 randomized controlled trials, Lochbaum et al. (2022) reviewed 13 meta-analyses examining the effects of various types of psychological interventions on sport performance. Although positive effects have been reported, effect sizes are heterogeneous and depend on the type of intervention (psychological or psychosocial), the techniques used (single or multi-component), and population characteristics (e.g., sex). Thus, there is a clear lack of evidence, and greater efforts need to be made to provide professional practitioners with support that is based on reliable research.

This issue is even more critical when focusing on elite performance. “Elite” can be defined in various ways, and the literature proposes different taxonomies (e.g., Swann et al., 2015; McKay et al., 2022). In particular, McKay et al. (2022) suggest a classification system that ranges from “sedentary” (Tier 0) to “world-class” (Tier 5). Tiers 4 and 5 describe groups of athletes who perform within 7% of world-record performance, with maximum or near-maximum training loads. All athletes in these tiers compete at international level, are ranked in the top 300 in their respective sport, and represent 0.003% of the global population. Their best performance relies on tiny, yet key details. Thus, it is crucial to better understand the impact of psychological interventions on performance in this specific population. Against this background, Brown and Fletcher (2017) evaluated the effects of sport psychology interventions on performance; their meta-analysis found that only one of the 35 included studies was conducted with “international” athletes. Thus, evidence regarding the effectiveness of sport psychology interventions on performance in elite athletes seems very weak.

## 2 Interventional research designs in elite sport: a brief review from 2012 to 2024

We conducted a brief review to understand how researchers evaluate and gather empirical evidence on the effectiveness of sport psychology interventions in elite athletes. Following the Preferred Reporting Items for Systematic Reviews and Meta-Analyses (PRISMA) guidelines, we searched SportDiscuss for studies, using the keywords: (Elite OR Expert) AND Athlete AND Sport AND (Intervention OR Program) AND Psychology NOT (Amateur OR Junior OR Adolescent). Studies were included if they met the following criteria: (a) they were interventional studies, (b) they were conducted with elite athletes (Tiers 4 or 5, as defined by McKay et al., 2022), and (c) they were published in peer-reviewed journals within the past 12 years. Initially, 652 articles were screened by an independent rater, resulting in the exclusion of 571 studies that were either meta-analyses, theses, or did not involve a psychological intervention. Of the 81 remaining articles, we assessed participant “eliteness” (score >8) using the taxonomy of Swann et al. (2015), which categorizes athletes as “semi-elite”, “competitive elite”, “successful elite”, or “world-class elite”. This assessment was based on a modified equation proposed by Gupta et al. (2017). Ultimately, only 11 studies were retained (see Table 1). Four designs were identified: single or multiple case studies (*n* = 6), pre-posttest with one group (*n* = 2), control group (*n* = 1), and randomized controlled trial (RCT) with a waitlist (*n* = 2). Measures were based on qualitative (*n* = 1), quantitative (*n* = 6), and mixed (*n* = 4) methods. Two studies measured objective effects on performance as a shooting score (Bortoli et al., 2012; Gröpel et al., 2020), while both studies used global performance (i.e., selection, medals) (Dupee et al., 2016; Takeuchi et al., 2023).

**Table 1:** Interventional studies with elite athletes published between 2012 and 2024.

**References**	**Sample**	**Sport**	**Intervention type**	**Program**	**Design**	**Data**
Bortoli et al. (2012)	15	Shooting	Psychological skills training	Multi-Action Plan	Multi-case study	Mixed
Demarzo et al. (2015)	1	Athletics	Mindfulness intervention	Mindfulness-Based Stress Reduction	Single case study	Mixed
Dupee et al. (2016)	5	-	Bio-neurofeedback	-	Multi-case study	Qualitative
Gröpel et al. (2020)	12	Shooting	Psychological skills training	Pre-shot routine intervention	Pre-posttest with one group	Quantitative
Haase et al. (2015)	7	BMX	Mindfulness intervention	Mindful Performance Enhancement, Awareness and Knowledge	Pre-posttest with one group	Quantitative
Jouper and Gustafsson (2013)	1	Shooting	Mindfulness intervention	Mindfulness and Qigong	Single case study	Quantitative
Lundgren et al. (2020)	21	Hockey	Mindfulness intervention	Acceptance and Commitment Therapy	Controlled trial	Quantitative
Macdougall et al. (2019)	18	Parasport	Mindfulness intervention	Mindfulness Acceptance Commitment	RCT with waitlist control group	Quantitative
Mehrsafar et al. (2019)	26	Sanda Wushu—Combat sport	Mindfulness intervention	Mindfulness-based intervention	RCT with waitlist control group	Quantitative
Takeuchi et al. (2023)	1	Surf	Psychological skills training	Mind-body training	Single case study	Mixed
Wood and Fletcher (2023)	1	Figure skating	Mindfulness intervention	Acceptance and Commitment Therapy	Single case study	Mixed

This brief review highlights that very few studies have been published over the past 12 years. We identify two main methodological boundaries, related to the specificities of elite performance, which limit interventional research studies. First, the population of elite athletes is very small, and training constraints mean that they are seldom available to take part in a time-consuming interventional study (McKay et al., 2022). In these conditions, implementing an RCT, recognized as the gold standard in psychology or health research, is not only almost impossible, but also inappropriate. Second, researchers face the difficulty of capturing the effects of psychological interventions on actual performance. The multifactorial nature of sport performance (where psychological, physical, and social factors are interwoven), and the complexity of the environment (luck, variable and uncertain conditions, etc.) make it very difficult to demonstrate the specific effects of psychological interventions. Moreover, the “plateau” phenomena is well-known among elite athletes, which means that any improvement is not only extremely tiny, but also not statistically significant (Lochbaum et al., 2022).

## 3 Methodological opportunities to strengthen the evidence base

Given these boundaries, sport psychology researchers must address the question of methodological standards in the field. They should also consider alternative ways to strengthen the evidence base and reinforce the effectiveness of sport psychology within this particular population.

### 3.1 Consider or reconsider alternative designs

Two types of design—single-case, and one group longitudinal—merit more attention as potential alternatives to the RCT. Based on an idiographic approach, single-case, or *n*-of-one designs are an opportunity to implement and evaluate the effects of a tailored intervention in one, or a few elite cases (e.g., one or a few athletes, coaches, or teams), while respecting real-life contextual and cultural specificities. Initially promoted in the domain of sport psychology by Hrycaiko and Martin (1996), and later Barker et al. (2013), the approach deserves renewed consideration. In particular, recent criticism of the RCT standard (Diener et al., 2022; Deaton and Cartwright, 2018; Cook, 2018) has driven substantial progress in the development of single-case designs and data analysis, and the approach could be a promising alternative to approximating causal inference in intervention research with elite athletes (Levin and Ferron, 2021; Manolov and Moeyaert, 2017).

One group longitudinal designs are another particularly interesting approach to overcoming the specific boundaries of research in elite sport. They consist in implementing an intervention in one group, and multiplying outcome variable measures at baseline, and throughout the intervention (Cece et al., 2022, 2023; Levillain et al., 2023). An ecological (e.g., a single-item definitional) approach, which emphasizes studying phenomena in their natural settings and accounting for the real-world constraints faced by elite athletes, can be particularly valuable. When combined with an innovative, multilevel statistical approach, such methods can provide new insights into the effects of interventions on psychological variables and performance.

### 3.2 Measuring effects on actual performance

As noted above, the effects of psychological interventions on actual performance have been insufficiently evaluated (Brown and Fletcher, 2017). However, this should be a primary goal, in order to validate professional practice. While scores, times, or distances may be meaningful indicators in some sports, they are not applicable in others. Building upon previous contributions on performance analysis (e.g., Araújo and Davids, 2016; Sarmento et al., 2022), it is important to develop and apply individual and team performance assessment methods in real-world conditions, based on an integrated, holistic approach (i.e., considering physical, technical, tactical, and psychosocial aspects). Rather than considering performance in terms of sports results (which depend on others' outcomes, luck, refereeing...), performance can be evaluated by assessing the quality of the execution of procedures or behaviors underlying sport performance. Notational analysis, athlete position analysis, or biomechanical analysis are all possible ways to measure performance, and these can also be combined.

### 3.3 Potential contributions of mixed methods

Mixed methods, which involve using both quantitative and qualitative methods within the same design, can help identify and explain the effects of interventions in elite populations (Mertens and Tarsilla, 2015). They allow researchers to take advantage of the strengths of both qualitative research (e.g., to describe the experience of athletes or coaches taking part in the intervention), and quantitative research, which uses outcome variables to determine effects (Creswell and Plano Clark, 2017). More specifically, a parallel and convergent mixed method might provide a more complete, contextual, and complex understanding of the effects, mechanisms, and underlying processes at play in a psychological intervention.

## 4 Conclusion

Three key challenges warrant attention to develop a reliable corpus of evidence regarding the effectiveness of psychological interventions in elite sport: (a) designing interventional studies with an optimal trade-off between rigor and relevance; (b) documenting mediating and moderating variables that strengthen effects of the intervention (e.g., the quality of the relation with the intervener, see Levillain et al., 2023); and (c) collaborating with practitioners to encourage implementation and transfer into real-life elite sport contexts. Addressing these three challenges may help researchers to improve both the quality and utility of interventional studies in elite sport.
